# Adult ileo-ileal intussusception from primary small bowel leiomyosarcoma: MRI diagnostic insights with corroborative histopathology

**DOI:** 10.1016/j.radcr.2026.04.073

**Published:** 2026-05-18

**Authors:** Karthik Rayasam, Belinda Sun, Salil Kalarn, Hazem H. Soliman, Hunter Jecius, Erol Bozdogan

**Affiliations:** a Department of Radiology and Imaging Sciences, The University of Arizona College of Medicine, Tucson, Arizona, USA; b Department of Pathology and Laboratory Medicine, The University of Arizona College of Medicine, Tucson, Arizona, USA

**Keywords:** Ileo-ileal intussusception, Small bowel leiomyosarcoma, MRI characterization, Histopathology correlation, Rare neoplasm

## Abstract

Adult intussusception is a rare condition and, unlike pediatric cases, is usually associated with an underlying structural lesion. Primary leiomyosarcoma of the small bowel is an exceptionally uncommon cause, with limited imaging correlations described in the literature. We report the case of a 60-year-old patient who presented with iron-deficiency anemia and intermittent abdominal pain. CT demonstrated ileo-ileal intussusception, prompting further evaluation with MRI. MRI confirmed a polypoid intraluminal mass serving as the lead point and demonstrated low signal intensity on T2-weighted imaging, diffusion restriction, and avid post-contrast enhancement. While these findings supported the presence of a solid neoplasm and facilitated confident identification and localization of the lead point, they were nonspecific and did not allow definitive tumor characterization. MRI therefore provided complementary anatomic and tissue-level information rather than a specific diagnosis. Given the high suspicion for a pathologic lead point, surgical resection was performed. Histopathologic examination established the diagnosis of leiomyosarcoma arising from the muscularis propria. Immunohistochemistry showed positivity for smooth muscle actin and negativity for c-Kit, DOG1, and S100, excluding gastrointestinal stromal tumor and other mesenchymal neoplasms. Pathology thus represented the diagnostic cornerstone. This case contributes to the limited literature describing MRI features of small bowel leiomyosarcoma presenting with adult intussusception and underscores the importance of a multimodal diagnostic approach, in which MRI serves as an adjunct to CT, while histopathology remains essential for definitive diagnosis.

## Introduction

Intussusception in adults is rare, constituting < 5% of intestinal obstructions, and typically occurs in the setting of a structural lead point [1]. Malignant tumors of the small bowel are uncommon, with adenocarcinoma, lymphoma, and gastrointestinal stromal tumor (GIST) being more frequently encountered. Primary leiomyosarcoma represents < 2% of gastrointestinal neoplasms and is an exceptionally rare etiology [2].

This case illustrates a rare instance of primary small bowel leiomyosarcoma presenting as intussusception, emphasizing MRI features and histopathologic correlation.

## Case report

A 60-year-old patient, evaluated for iron deficiency anemia and recurrent abdominal pain, underwent CT and MRI for further investigation.

### CT and MRI findings

**Fig. 1:**
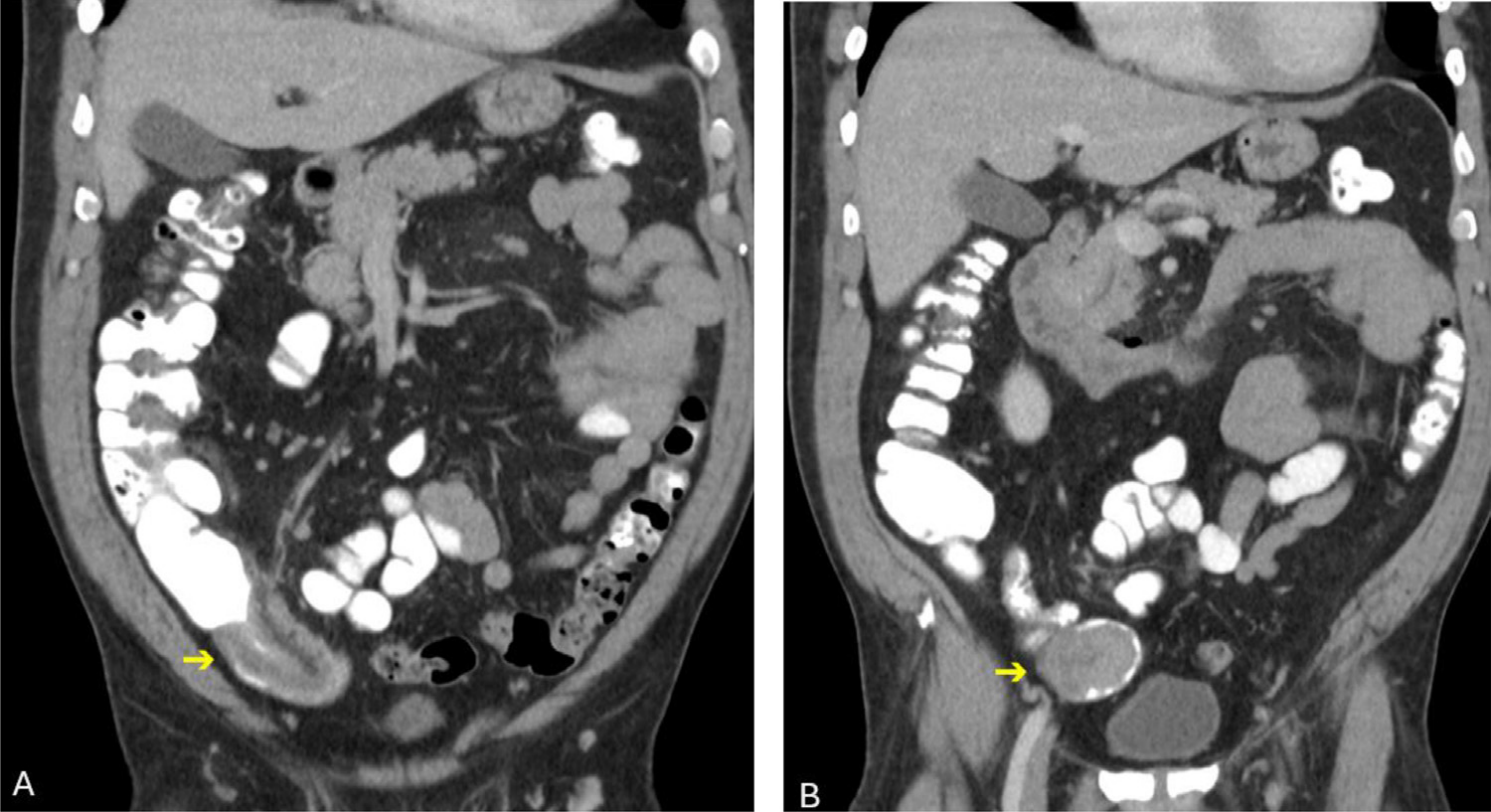
Coronal contrast-enhanced CT of the abdomen demonstrates ileo-ileal intussusception in the right lower quadrant (A). The intraluminal polypoid lead-point mass is clearly visualized (arrow, B), outlined by a circumferential rim of contrast*,* which highlights its intraluminal origin.

Initial contrast-enhanced CT (Figs. 1A and B) demonstrates an ileo-ileal intussusception in the right lower quadrant. Within the intussuscepted loop (Fig. 1B), there is a well-circumscribed, homogeneously enhancing intraluminal mass with a thin circumferential rim of contrast, suggesting an intraluminal origin.

**Fig. 2:**
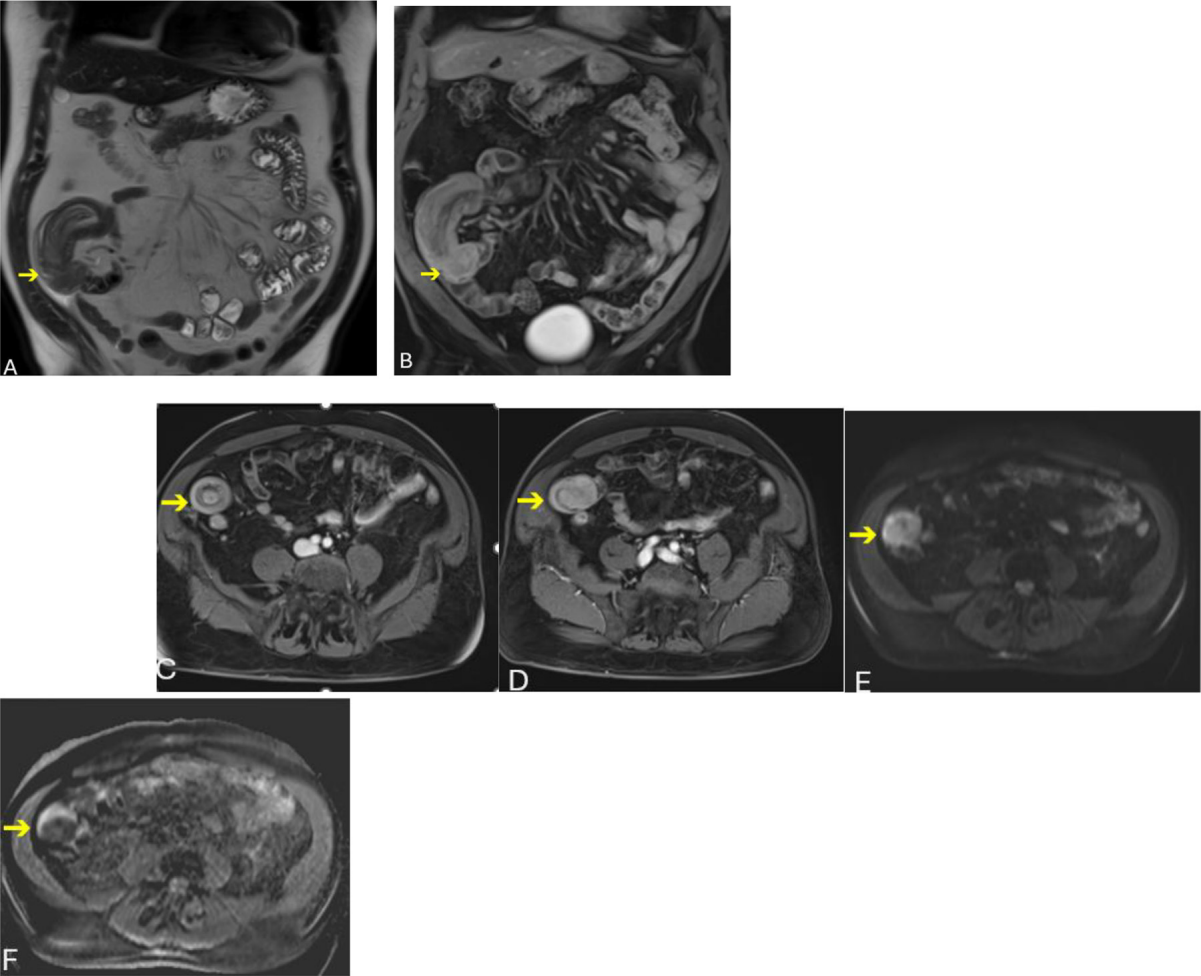
MRI of the abdomen demonstrates ileo-ileal intussusception. (A) Coronal T2-weighted image shows the intussuscepted ileal loops with a T2 hypointense polypoid mass at the leading edge (arrow). (B) Coronal fat-suppressed post-contrast T1-weighted image demonstrates avid enhancement of the lead-point mass (arrow). (C) Axial fat-suppressed post-contrast T1-weighted image illustrates the classic *“target sign”* of intussusception (arrow). (D) Axial fat-suppressed post-contrast T1-weighted image further delineates the enhancing intraluminal mass at the lead point (arrow). (E) and (F) Axial DWI/ADC map images demonstrate intense diffusion restriction (arrow in E) and low ADC (arrow in F), indicative of viable hypercellular tumor.

Subsequent MRI of the abdomen, performed using an MR enterography protocol on a Siemens 1.5T system with a phased-array body coil after administration of 24 oz of oral water, included multiplanar T2-weighted SPAIR sequences, diffusion-weighted imaging (b = 0 and 800 s/mm²), pre-contrast T1-weighted imaging (1.2-mm slice thickness), and dynamic post-contrast imaging following intravenous administration of 11 mL gadobenate dimeglumine, with arterial (22 seconds), portal venous (∼70 seconds), and delayed (∼5 minutes) phases. This examination (Figs. 2A–F) confirms an ileo-ileal intussusception involving the distal ileum just proximal to the ileocecal junction, extending approximately 20 cm, without upstream small-bowel dilatation. A 4.5-cm polypoid intraluminal mass is identified at the lead point, appearing T2-hypointense (Fig. 2A) and demonstrating avid post-contrast enhancement (Figs. 2B and D), with a characteristic target-type configuration of the intussuscepted bowel (Fig. 2C). Diffusion-weighted imaging demonstrates restricted diffusion with corresponding low ADC values (Figs. 2E and F).

### Surgical management

The patient underwent laparoscopic exploration, which identified an ileo-ileal intussusception involving the mid ileum. Segmental small bowel resection including the intussuscepted segment was performed, followed by primary stapled side to side anastomosis. Gross examination of the resected specimen revealed an intraluminal polypoid lesion. The procedure was completed without complication.

### Histopathology

**Fig. 3:**
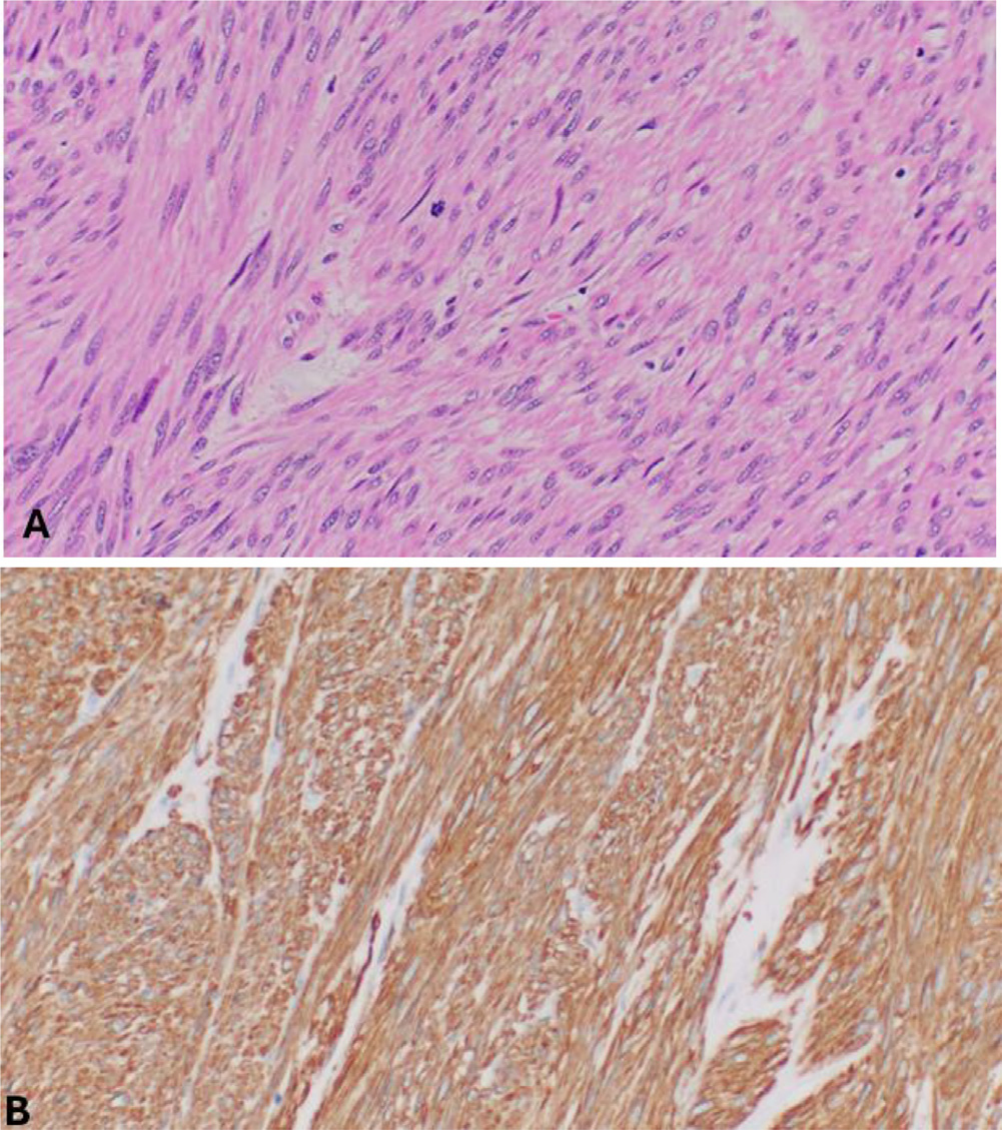
Leiomyosarcoma histology. (A) The leiomyosarcoma shows spindle cell with nuclear atypia and high mitotic activity. (B) Immunohistochemistry shows that the tumor cells are strongly positive for SMA.

Examination of the resected specimen revealed a spindle cell neoplasm arising from the muscularis propria. Tumor cells showed nuclear atypia and frequent mitoses. Immunohistochemistry was positive for smooth muscle actin (SMA) and negative for c-Kit, DOG1, S100, and ALK1. The Ki-67 proliferation index was up to 40%. Resection margins were negative. These findings were diagnostic of leiomyosarcoma (FNCLCC grade 2) (Figs. 3A and B).

### Follow up and clinical course

Surveillance CT of the chest and MRI of the abdomen and pelvis demonstrated no evidence of metastatic disease or recurrence. The patient continues routine oncologic follow-up with standard surveillance. The clinical timeline is summarized in Table 1.

**Table 1:** Clinical timeline.

Time point	Clinical event
∼ 1 month before surgery	Evaluation for iron-deficiency anemia and intermittent abdominal pain. Upper endoscopy was normal. CT abdomen/pelvis and MR enterography demonstrated a small-bowel mass with associated ileo-ileal intussusception.
Day 0 (Surgery)	Laparoscopic-assisted small bowel resection with stapled side-to-side anastomosis.
Day +11 (Pathology)	Leiomyosarcoma of the small bowel, 4.5 cm, FNCLCC grade 2, arising from the muscularis propria, margins negative, pathologic stage pT1; Ki-67 up to 40%.
∼ 3 months postoperatively	Surveillance imaging (CT chest and MRI abdomen/pelvis) demonstrated no evidence of local recurrence or metastatic disease.
Present	Postoperative oncologic surveillance is currently ongoing under the care of medical oncology.

## Discussion

Adult intussusception is uncommon, accounting for fewer than 5% of cases of intestinal obstruction, and when it involves the small bowel, it is frequently associated with an underlying structural lead point, most often a neoplasm [1]. While adenocarcinoma, lymphoma, and GIST represent the most reported etiologies, primary leiomyosarcoma of the gastrointestinal tract is exceedingly rare, accounting for less than 2% of gastrointestinal malignancies [2].

Adult intussusception in the small bowel is most evaluated using computed tomography (CT), which reliably demonstrates the characteristic bowel-within-bowel configuration and often identifies an underlying lead-point lesion [3]. Prior descriptions of adult intussusception caused by neoplasms have relied predominantly on CT imaging. Gayer et al. [3] emphasized CT’s role in identifying causative intraluminal or mural masses, with most reported lead points representing adenocarcinoma, lymphoma, or GIST. Published reports specifically addressing small bowel leiomyosarcoma presenting as intussusception, including those summarized by Bouassida et al. [4], have primarily described CT findings, typically nonspecific soft tissue masses serving as lead points. Kim et al. [5] similarly highlighted the utility of CT in identifying the presence and cause of adult intussusception, with leiomyosarcoma remaining an uncommon etiology.

Magnetic resonance imaging (MRI) has been discussed more broadly in the evaluation of small bowel neoplasms, particularly by Masselli et al. [6] and Jasti and Carucci [7], who detailed MRI characteristics of entities such as GIST, adenocarcinoma, and lymphoma. However, these reviews do not specifically describe leiomyosarcoma presenting with intussusception, nor do they provide dedicated MRI correlations for this rare entity.

To our knowledge, there are no prior published reports describing the MRI appearance of small bowel leiomyosarcoma presenting as intussusception. In the present case, MRI demonstrated an intraluminal polypoid mass with relatively low T2 signal intensity, avid post-contrast enhancement, and diffusion restriction. While these imaging features are nonspecific and overlap with other malignant small bowel tumors, their documentation in a histopathologically confirmed leiomyosarcoma adds novel imaging correlation. Importantly, the association between imaging appearance and tumor subtype was established retrospectively following surgical resection and pathologic confirmation.

This case therefore extends the existing CT-based literature on adult intussusception [[3], [4], [5]] by providing previously unreported MRI findings of small bowel leiomyosarcoma, contributing incremental value to the imaging characterization of this rare diagnosis.

## Conclusion

Primary small bowel leiomyosarcoma is an exceptionally rare cause of adult intussusception. Cross-sectional imaging is essential for identifying intussusception and detecting an underlying lead point; however, imaging findings remain largely nonspecific. In this context, MRI serves as a valuable complementary tool to CT by improving lesion conspicuity, delineating intraluminal morphology, and aiding preoperative assessment, rather than enabling definitive tumor characterization. Radiologic–pathologic correlation remains fundamental for establishing the final diagnosis. This unusual case, combining MRI depiction of the lead point with histopathologic confirmation, adds to the limited existing literature and provides an important educational reference for radiologists and clinicians involved in the evaluation of adult intussusception.

## Learning points

• Adult intussusception is rare and frequently associated with a structural neoplastic lead point.
• Leiomyosarcoma, though rare, should be considered in the differential of intramural small bowel masses.
• MRI provides high resolution tissue characterization, revealing features not always apparent on CT.
• Immunohistochemistry distinguishes leiomyosarcoma (SMA-positive, c-Kit/DOG1-negative) from GIST, driving therapeutic decisions.
• Surgical resection remains the cornerstone of management due to limited systemic treatment options.

## Declaration of generative AI and AI-assisted technologies in the writing process

During the preparation of this manuscript, the authors used ChatGPT (OpenAI) solely to improve language and readability. After using this tool/service, the authors reviewed and edited the content as needed and take full responsibility for the content of the published article.

## Authors’ contributions

All authors were involved in patient care, data acquisition, manuscript writing, and critical revision. All authors have approved the final version of the manuscript.

## Patient consent

A written informed consent was obtained from the patient for the publication of this case report.
